# Mechanoregulation of MSC spheroid immunomodulation

**DOI:** 10.1063/5.0184431

**Published:** 2024-03-01

**Authors:** Victoria L. Thai, Sabrina Mierswa, Katherine H. Griffin, Joel D. Boerckel, J. Kent Leach

**Affiliations:** 1 Department of Orthopaedic Surgery, UC Davis Health, Sacramento, California 95817, USA; 2 Department of Biomedical Engineering, University of California, Davis, Davis, California 95616, USA; 3 School of Veterinary Medicine, University of California, Davis, Davis, California 95616, USA; 4 Department of Orthopaedic Surgery, University of Pennsylvania, Philadelphia, Pennsylvania, 19104, USA

## Abstract

Mesenchymal stromal cells (MSCs) are widely used in cell-based therapies and tissue regeneration for their potent secretome, which promotes host cell recruitment and modulates inflammation. Compared to monodisperse cells, MSC spheroids exhibit improved viability and increased secretion of immunomodulatory cytokines. While mechanical stimulation of monodisperse cells can increase cytokine production, the influence of mechanical loading on MSC spheroids is unknown. Here, we evaluated the effect of controlled, uniaxial cyclic compression on the secretion of immunomodulatory cytokines by human MSC spheroids and tested the influence of load-induced gene expression on MSC mechanoresponsiveness. We exposed MSC spheroids, entrapped in alginate hydrogels, to three cyclic compressive regimes with varying stress (L) magnitudes (i.e., 5 and 10 kPa) and hold (H) durations (i.e., 30 and 250 s) L5H30, L10H30, and L10H250. We observed changes in cytokine and chemokine expression dependent on the loading regime, where higher stress regimes tended to result in more exaggerated changes. However, only MSC spheroids exposed to L10H30 induced human THP-1 macrophage polarization toward an M2 phenotype compared to static conditions. Static and L10H30 loading facilitated a strong, interlinked F-actin arrangement, while L5H30 and L10H250 disrupted the structure of actin filaments. This was further examined when the actin cytoskeleton was disrupted via Y-27632. We observed downregulation of YAP-related genes, and the levels of secreted inflammatory cytokines were globally decreased. These findings emphasize the essential role of mechanosignaling in mediating the immunomodulatory potential of MSC spheroids.

## INTRODUCTION

Tissue repair is a dynamic process involving direct and indirect crosstalk among cells via chemokines, growth factors, and signaling pathways triggered by the environment (e.g., adhesion ligands, nutrient gradients, substrate stiffness, and mechanical stimuli).^1^ This understanding has led to tissue engineering strategies that guide cell fate in an effort to regenerate damaged tissues. Mesenchymal stromal cells (MSCs) are among the most frequently used cell type for regenerative medicine due to their high proliferative capacity, multilineage potential, and potent bioactive secretome. The MSC secretome elicits key events required for tissue repair such as initiating vascularization through secreted pro-angiogenic factors (e.g., VEGF, PDGF, bFGF, and IL-6) and regulating inflammation through secreted immunomodulatory chemokines and cytokines (e.g., PGE_2_, IL-4, IL-6, IL-8, and IL-10).^2^ The secretion of these cytokines and growth factors is enhanced when MSCs are simply aggregated into spheroids.^3–6^ Moreover, spheroids exhibit improved cell viability, persistence, and retention of endogenous ECM compared to monodisperse MSCs, motivating their continued study for cell-based therapies.^7,8^

Biomaterials, and especially hydrogels, are commonly used as cell carriers because they increase cell retention and localization at the implantation site and can direct cell behavior.^9^ Naturally derived alginate hydrogels are frequently employed to direct MSC function due to their tunability of stiffness, adhesivity, and viscoelasticity.^10^ For example, MSCs in softer alginate hydrogels (∼2 kPa) increased secretion of CCL2 and IL-6, two factors involved in monocyte recruitment, compared to stiffer (∼35 kPa) hydrogels.^11^ Furthermore, viscoelastic alginate gels enhanced the osteogenic potential of MSC spheroids compared to more elastic alginate gels.^12^ Hence, we leveraged alginate hydrogels to investigate the influence of the biophysical microenvironment on cytokine secretion by MSC spheroids.

Mechanosignaling is a key mediator of how cells sense their microenvironment, resulting in differentiation, migration, and proliferation.^13^ Monodisperse MSCs exhibited increased cytokine production when subjected to compression.^14^ In particular, MSCs secreted more vascular endothelial growth factor (VEGF), a potent angiogenic factor, when mechanically stimulated compared to unstimulated cells. Other studies reported an upregulation of additional proangiogenic factors, including TGF-β1 and placental growth factor (PIGF), when mechanically loaded.^15,16^ In other work, the magnitude and duration of mechanical stimulation modulated MSC osteogenic potential.^17,18^ However, these systems utilized hydrostatic and pneumatic pressures to apply mechanical loads, which do not emulate *in vivo* conditions, as these forces are not physically in contact with the construct. Systems that capture this physical aspect with platens have only studied monodisperse MSCs to date, leaving the effects of compressive load on MSC spheroids unknown.^18–20^ The duration of stimulation also influences MSC behavior, as shorter exposure times (i.e., 2.5 h/day) with cyclic compressions at a frequency of 1 Hz promoted more Sox9 and collagen type II expression compared to longer times (i.e., 4 h/day).^21^ However, there have been no studies to investigate the effects of both load amplitude and duration on MSC spheroid behavior.

We hypothesized that cyclic compressive load and loading regime (i.e., load magnitude and duration) modulates the immunomodulatory potential of alginate entrapped MSC spheroids characterized by changes in cytokine production, gene expression, and macrophage polarization. We utilized a compressive loading bioreactor that generates controlled uniaxial compressions to interrogate the influence of compressive load on MSC spheroid behavior. We explored the mechanism of action regulating the secretory potential of MSC spheroids. This study describes the influence of compressive loading on the immunomodulatory potential of MSC spheroids and emphasizes the importance of cyclic compressive load for tissue repair.

## RESULTS

### Mechanical characterization of alginate hydrogel

**FIG. 1.**
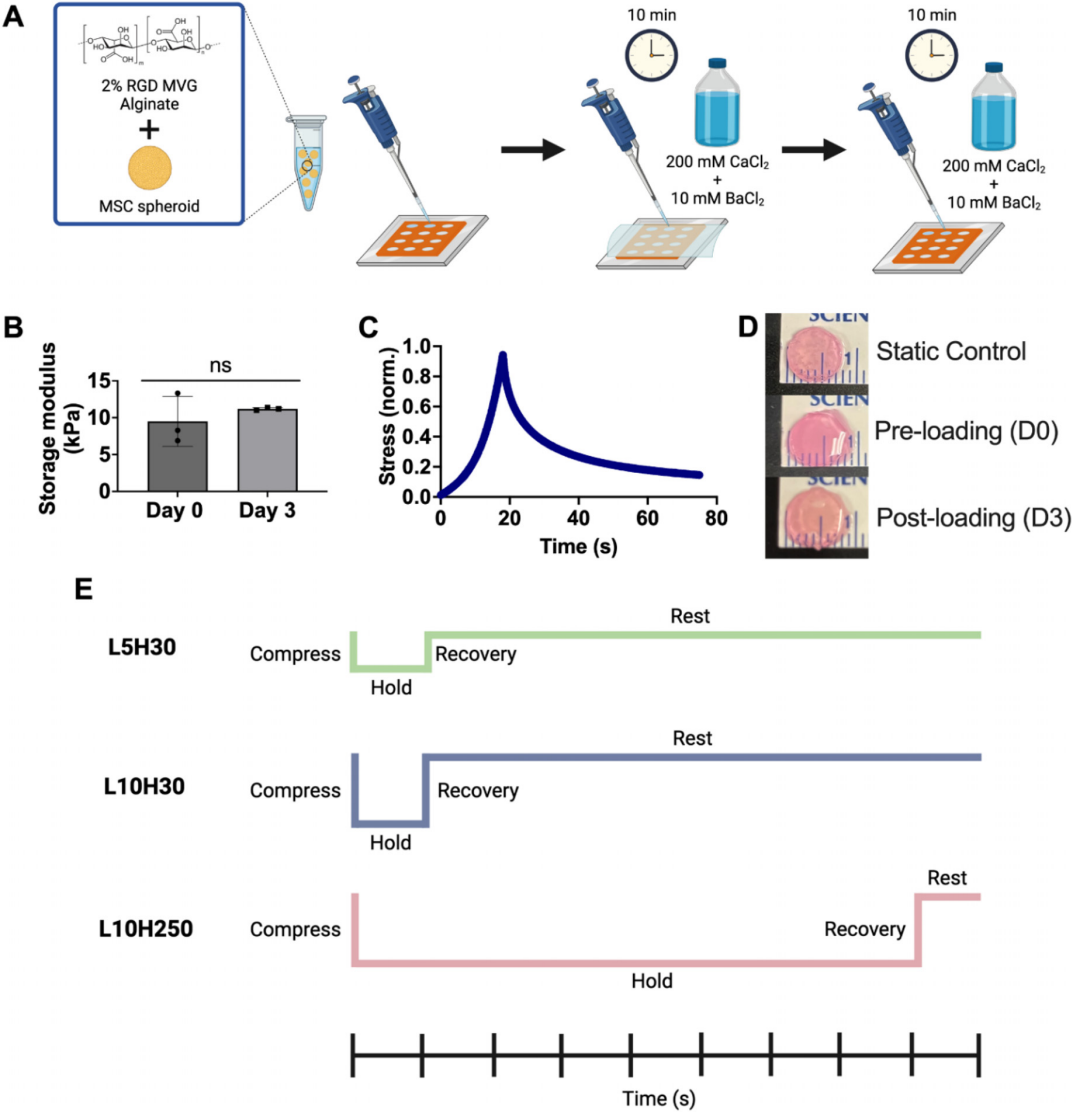
Schematic of synthesis and mechanical characterization of alginate hydrogels. (a) Schematic of spheroid encapsulation in RGD-modified alginate. (b) Storage modulus of the gels pre- (D0) and post-swelling (D3) was unchanged in static culture (n = 3). (c) Representative stress relaxation curve used for alginate gels on day 1 after reaching swelling equilibrium. (d) Morphological view of alginate gels in static culture and pre- (D0) and post-loading (D3) in the bioreactor. (e) Illustration of bioreactor loading regimes (L5H30, L10H30, and L10H250) with varying load (L) magnitudes (i.e., 5 kPa, 10 kPa) and hold (H) durations (i.e., 30 s, 250 s). Each line segment represents 30 s intervals. All cycle times were equivalent in total duration (5 min 30 s). L5H30—5 kPa load, 30 s hold; L10H30—10 kPa load, 30 s hold; L10H250—10 kPa load, 250 s hold.

MSC spheroids were encapsulated in RGD-modified alginate hydrogels and cultured in static or dynamic conditions [Fig. 1(a)]. The storage modulus (∼10 kPa) remained unchanged after 3 days in culture [Fig. 1(b)]. The average stress relaxation time of the alginate hydrogels was 28.1 s, similar to other reported stress relaxation times for MVG alginate [Fig. 1(c)].^12,22^ We confirmed that there was less than 10% change in stress in loaded gels over the first 48 h, which was calculated by dividing the magnitude of the applied load by the area of the hydrogel at 24 and 48 h (data not shown). From these data, compressive stresses of 5 and 10 kPa and hold times of 30 and 250 s were selected to generate three loading regimes—5 kPa load with 30 s hold (L5H30), 10 kPa with 30 s hold (L10H30), and 10 kPa with 250 s hold (L10H250) [Fig. 1(e)] (Table I). The loading times were selected to be similar or substantially longer than the gel relaxation time to differentially stimulate entrapped cells with applied stresses. Gross morphological images of spheroid-entrapped alginate gels revealed a 25% increase in gel diameter after compression with 5 and 10 kPa compared to pre-loaded gels (8 mm) [Fig. 1(d)]. Static culture gels remained unchanged in diameter.

**TABLE I.** Bioreactor cyclic compressive loading regimes.

Loading regime	Stress (kPa)	Compression (s)	Hold duration (s)	Recovery (s)	Rest (s)
L5H30	5	30	30	30	240
L10H30	10	30	30	30	240
L10H250	10	30	250	30	20

### Cyclic compressive load modulates MSC spheroid diameter

**FIG. 2.**
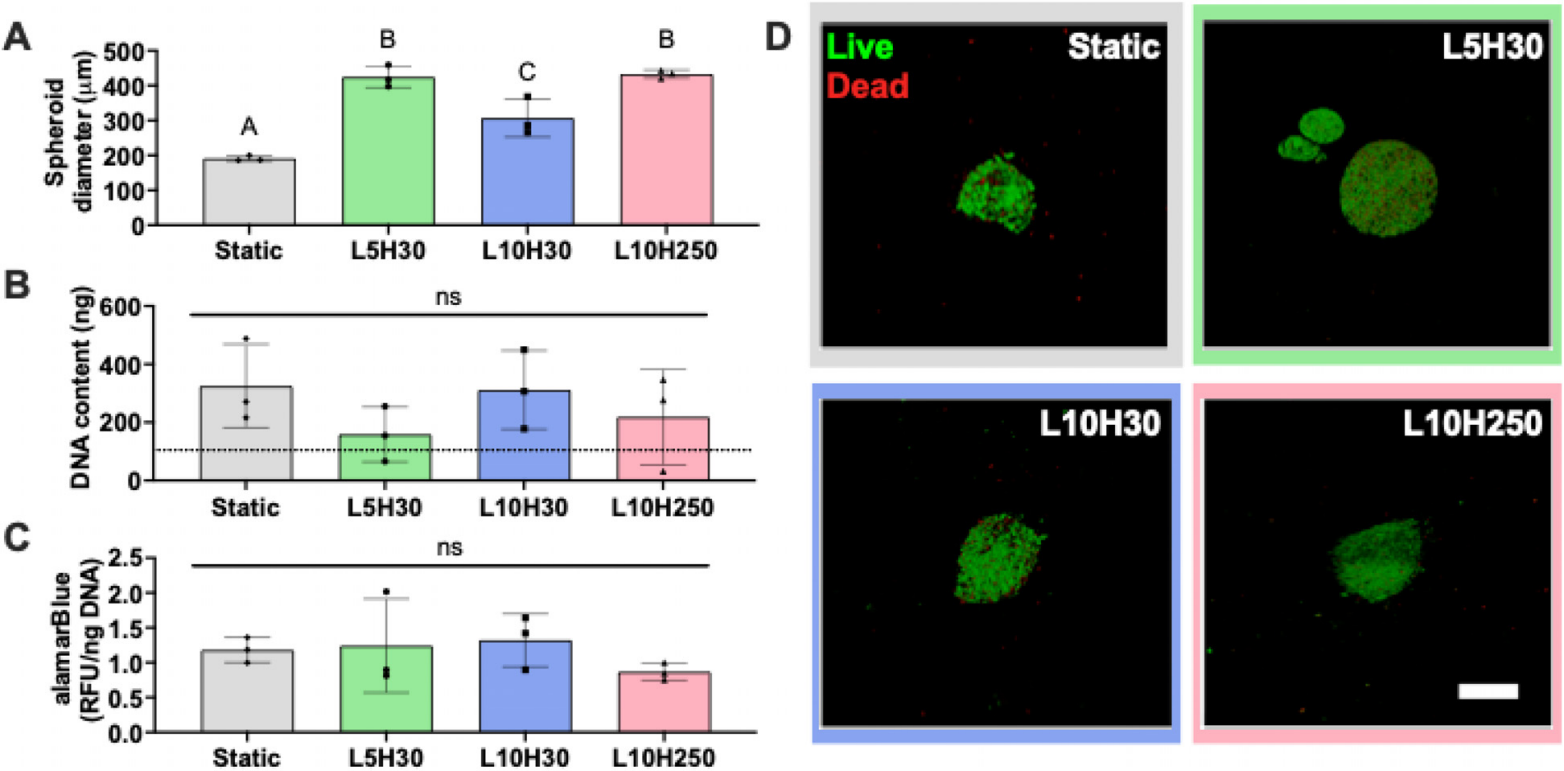
Cyclic compressive mechanical load increases MSC spheroid diameter but not total DNA, metabolic activity, and cell viability. (a) MSC spheroid diameter increased under compressive mechanical loads (L5H30, L10H30, and L10H250) compared to static culture on day 3. (b) Total DNA and (c) alamarBlue staining of spheroids were unchanged in static or dynamic culture. The dotted black line represents total DNA content of spheroids immediately after entrapment. (d) Live/dead staining revealed that cells were viable in all culture conditions. Scale bar represents 200 *μ*m. Data are mean ± SD (n = 3). Different letters denote statistical differences. L5H30—5 kPa load, 30 s hold; L10H30—10 kPa load, 30 s hold; L10H250—10 kPa load, 250 s hold.

MSC spheroid-loaded alginate hydrogels were cultured under cyclic compressive load or in static conditions to investigate the influence of mechanical loading on the bioactivity of MSC spheroids. After 3 days of culture, spheroids in compression-treated gels had larger diameters compared to spheroids in static groups. L5H30 (425 ± 31 *μ*m, *p* < 0.001), L10H30 (307 ± 54 *μ*m, *p* < 0.01), and L10H250 (433 ± 12 *μ*m, *p* < 0.001) had 2.2-fold, 1.6-fold, and 2.3-fold larger diameters than spheroids in static control (191 ± 8 *μ*m), respectively [Fig. 2(a)]. MSC spheroids contained 105 ± 32 ng DNA immediately after entrapment in the alginate (*n* = 3). On day 3, spheroids in L10H30 groups (312 ± 136 ng DNA) exhibited similar DNA content as spheroids in static conditions (325 ± 144 ng DNA), suggesting that L10H30 conditions supported similar proliferation as static culture. Spheroids in L5H30 (159 ± 95 ng DNA) and L10H250 (232 ± 144 ng DNA) conditions contained less DNA, although not significantly different [Fig. 2(b)]. This suggests that cyclic mechanical loading is not detrimental for proliferation of MSC spheroids in alginate hydrogels. Similarly, there were no differences in alamarBlue staining among the groups, suggesting that mechanical load does not suppress metabolic activity of MSC spheroids [Fig. 2(c)]. Cells remained viable for all groups as evidenced by Live/Dead staining [Fig. 2(d)]. Overall, while an increase in spheroid diameter was observed for mechanically stimulated groups, this did not affect proliferation, metabolic activity, and cell viability of MSC spheroids compared to those in static conditions, emphasizing that compressive load is not detrimental to MSC function.

### Magnitude and duration of mechanical load influences MSC cytokine production

**FIG. 3.**
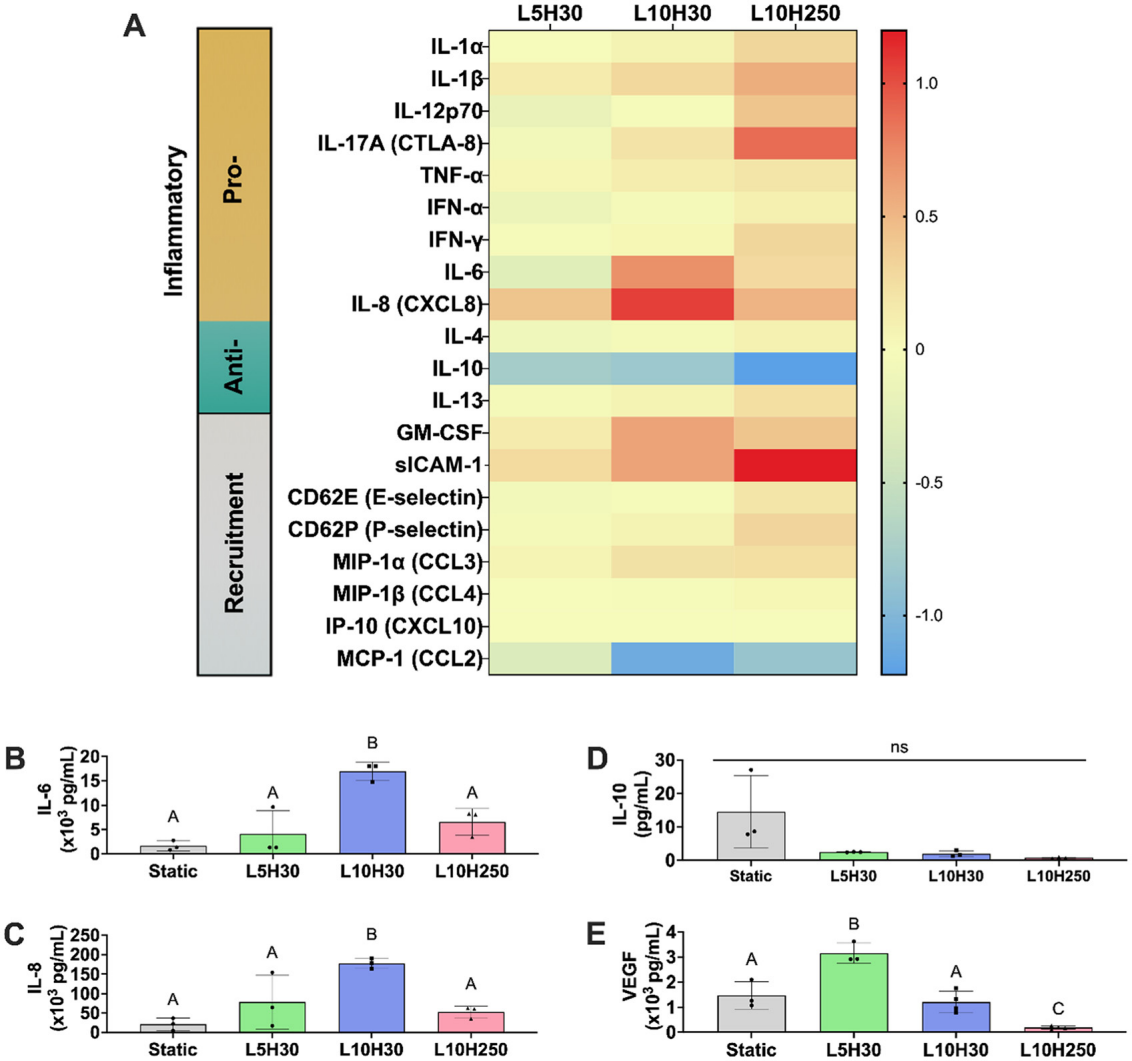
Compressive loading regime modulates MSC spheroid immunomodulatory and angiogenic secretome. (a) Results of a quantitative 20-plex Luminex assay reflected in the heat map. Data are normalized to static culture controls. The scale bar represents fold changes in cytokine/chemokine content where 0 represents no change (yellow), positive values represent an upregulation with respect to static controls (red), and negative values represent a downregulation with respect to static controls (blue). (b) Total IL-6, (c) IL-8, (d) IL-10, and (e) VEGF concentration secreted by MSC spheroids in static and dynamic culture. Data are mean ± SD (n = 3–4). Different letters denote statistical differences. L5H30—5 kPa load, 30 s hold; L10H30—10 kPa load, 30 s hold; L10H250—10 kPa load, 250 s hold.

To determine the influence of cyclic compressive load on the MSC secretome, we characterized the secretory profiles of spheroids in alginate hydrogels under static and dynamic culture using a multiplex cytokine assay. Of the analytes examined, IL-1β, IL-8, GM-CSF, and sICAM-1 secretion were prominently upregulated, while IL-10 secretion was downregulated by spheroids under L5H30 conditions compared to static culture [Fig. 3(a)]. Interestingly, larger compressive loads and longer hold times (L10H30 and L10H250) resulted in greater fold changes in pro-inflammatory, anti-inflammatory, and recruitment cytokines and chemokines, evidenced by the more diverse and complex secretory profiles. L10H30-loaded MSC spheroids upregulated IL-6, IL-8, GM-CSF, and sICAM-1 and downregulated IL-10 and CCL2 compared to static controls. With longer hold times (L10H250), MSC spheroids secreted higher levels of IL-1β, IL-17A, IL-8, and sICAM-1 and less IL-10 and CCL2 compared to static groups.

Secretion of pro- and anti-inflammatory cytokines by MSC spheroids was dependent on compressive load magnitude and duration. IL-6 and IL-8, two important pro-inflammatory cytokines, were significantly increased in L10H30 conditions compared to static, L5H30, and L10H250 conditions [Figs. 3(b) and 3(c)], but the magnitude of these factors decreased with increasing hold time. Overall, MSC spheroids exposed to compression (L5H30, L10H30, and L10H250) exhibited 2.5-, 10.3-, and fourfold more IL-6 and 3.7-, 8.4-, and 2.5-fold more IL-8 compared to static controls, respectively. Conversely, IL-10, a potent anti-inflammatory cytokine, was generally downregulated in MSC spheroids under compressive load [Fig. 3(d)]. These data demonstrate that compression increased several common pro-inflammatory cytokines, while IL-10, a potent anti-inflammatory cytokine, was decreased with compression.

We also assessed changes in the pro-angiogenic potential of MSC spheroids under different loading conditions. L5H30 promoted 2.1-, 2.6-, and 17-fold more VEGF secretion from MSC spheroids compared to spheroids in static, L10H30, and L10H250 conditions, respectively [Fig. 3(e)]. Strikingly, while L5H30 enhanced VEGF secretion, larger compressive loads and longer durations (L10H250) downregulated VEGF secretion compared to spheroids in static culture, establishing that VEGF secretion is dependent on load magnitude and hold time. Overall, these data emphasize that the application of a cyclic compressive load modulates the secretion of immunomodulatory and pro-angiogenic factors from MSC spheroids compared to static culture.

### Compressive loading disrupts the MSC actin cytoskeleton

**FIG. 4.**
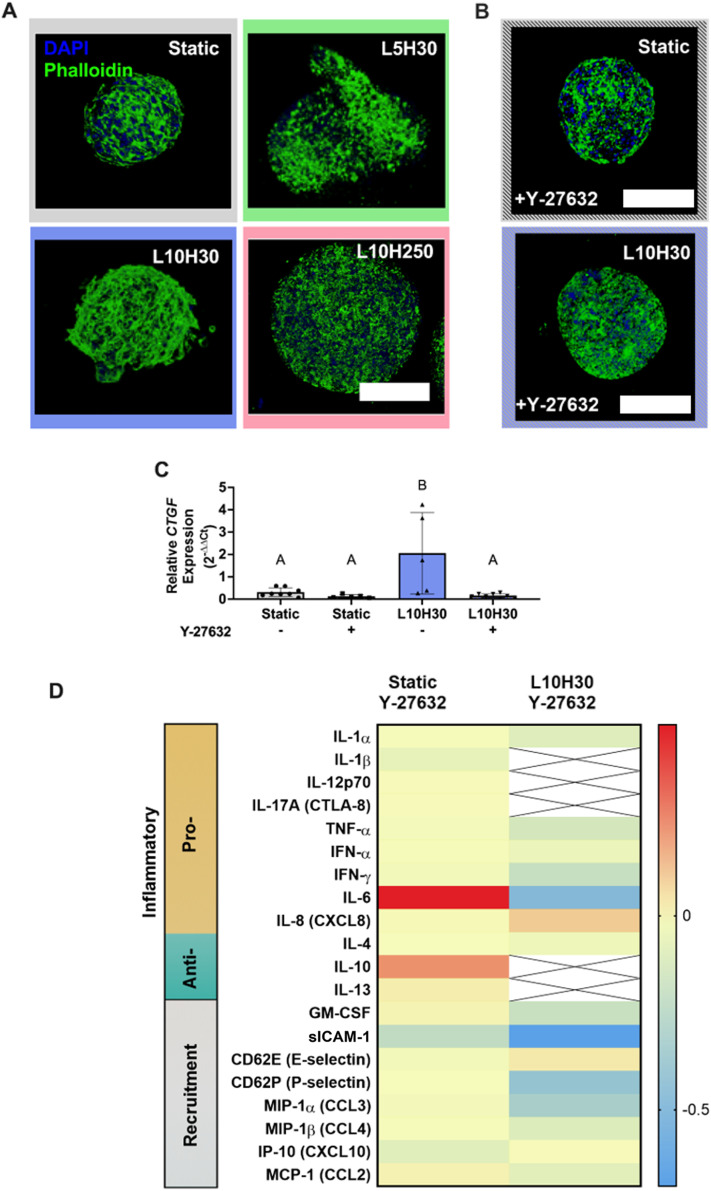
MSC spheroids are mechanoresponsive to cyclic compressive load through the actin cytoskeleton. (a) Spheroids stained with phalloidin (green) for actin and DAPI (blue) for cell nuclei demonstrated robust actin filament structure in static and L10H30 groups on day 3. Scale bar represents 200 *μ*m. (b) Treatment with 10 *μ*M Y-27632 for 24 h disrupted spheroid actin filament structure where phalloidin (green) was stained for actin and DAPI (blue) was stained for cell nuclei in static and L10H30 groups. Scale bars represent 200 *μ*m. (c) Compressive loading enhanced *CTGF* expression in MSC spheroids in L10H30 compared to static culture (*n* = 3–8). *GAPDH* was used as the housekeeping gene and day 0 spheroids were used as the control. (d) A heat map shows the results of a quantitative 20-plex Luminex assay with reactivity for pro- and anti-inflammatory and inflammatory cell recruitment human analytes. Data are normalized either to untreated static or untreated L10H30 groups and represent log fold change. The scale bar represents fold changes in cytokine/chemokine content where 0 represents no change (yellow), positive values represent an upregulation with respect to the corresponding controls (red), and negative values represent a downregulation with respect to the corresponding controls (blue). Crossed out boxes denote secretory levels for those analytes were undetectable. Compared to the respective controls, static Y-27632-treated groups demonstrated minimal changes in secretory potential, while L10H30 Y-27632 groups exhibited global downregulation of the analytes examined. L5H30 – 5 kPa load, 30 s hold; L10H30—10 kPa load, 30 s hold; L10H250—10 kPa load, 250 s hold.

We further assessed the effect of compressive loading on the cytoskeleton via actin filament (F-actin) staining [Fig. 4(a)]. MSCs in static groups possessed thick aligned filaments throughout the spheroid with some cellular protrusions into the surrounding hydrogel. Spheroids loaded with L10H30 also exhibited thick filament formation throughout the spheroid, but the bundles of F-actin were organized into an interconnected meshwork. Strikingly, when the compressive load was decreased (L5H30) or hold time was increased (L10H250), the F-actin networks present in the static and L10H30 groups were disrupted, as the F-actin structure was no longer visible. This demonstrates that the magnitude and hold time of mechanical load regulates the assembly and structure of actin filaments. Higher compressive load and shorter hold time facilitated the formation of thicker and more robust stress fibers.

### MSC spheroids are mechanoresponsive via cytoskeletal machinery

The actin cytoskeleton is important for maintaining cell shape, structure, and migration, and functions as a mechanosensor of external forces.^23^ To test for a role of cytoskeletal mechanotransduction, we evaluated signaling downstream of the mechanosensitive transcriptional regulator, Yes-associated protein (YAP). YAP is sequestered in the cytosol under conditions of low cytoskeletal tension but translocates to the nucleus upon cytoskeletal activation.^24^ In the nucleus, YAP binds to and co-regulates the transcriptional activity of other transcription factors, resulting in mechanotransduction. *CTGF* is a YAP-TEAD target gene that encodes the matricellular growth factor, Connective Tissue Growth Factor. We therefore hypothesized that compressive loading would induce upregulation of *CTGF* expression compared to static culture conditions. As expected, spheroids under L10H30 stimulation exhibited 11-fold more *CTGF* expression relative to static spheroids, respectively [Fig. 4(c)].

Next, we used Y-27632, an inhibitor of Rho kinase (ROCK), to disrupt the actin cytoskeleton.^25^ We hypothesized that Y-27632 treatment would downregulate *CTGF* expression in MSC spheroids in L10H30 culture with minimal effect on those in static culture. Due to the load-related disruption in actin filaments exhibited in L5H30 and L10H250 culture [Fig. 4(a)], we focused on L10H30 as our dynamic loading condition. Actin filament staining confirmed the disruption of the actin cytoskeleton by Y-27632 in both static and L10H30 culture conditions [Fig. 4(b)]. We further confirmed cells remained viable after 24 h treatment with Y-27632 by live/dead stain (data not shown). L10H30-loaded MSC spheroids exhibited 14-fold less *CTGF* expression when treated with Y-27632, while there were no differences in expression for the static culture groups [Fig. 4(c)]. Together, these data suggest that MSC spheroids are responsive to cyclic compressive load through the actin cytoskeleton.

### Y-27632 treatment dampens MSC spheroid secretion of inflammatory cytokines

We further explored the effect of Y-27632 treatment on the secretome of MSC spheroids cultured in static or L10H30 conditions by characterizing the inflammatory secretory profiles using a multiplex cytokine assay. Treatment with the inhibitor impeded the overall analyte secretion from MSC spheroids [Fig. 4(d)]. We observed a 32% decrease in total secretion for spheroids treated with Y-27632 in static conditions (18 438 pg/ml) compared to untreated static spheroids (27 038 pg/ml) (supplementary material Table 1). Similarly, spheroids loaded with L10H30 had a 31% reduction in total secretion when treated with Y-27632 (2437 pg/ml) compared to those in the untreated L10H30 group (3547 pg/ml). Of notable distinction, we detected an 87% reduction in total analyte secretion for treated L10H30 compared to untreated static groups, further emphasizing the response to mechanical cues.

Of the analytes examined, Y-27632-treated spheroids in static culture exhibited similar secretory levels as those in untreated static culture with a slight downregulation in sICAM-1 and CXCL10 [Fig. 4(d)]. When comparing Y-27632-treated L10H30 spheroids, we observed an overall downregulation in pro-inflammatory, anti-inflammatory, and recruitment analyte secretion, particularly IL-6, sICAM-1, P-selectin, and CCL3. Y-27632-treated L10H30 spheroids exhibited reductions all detectable analytes excluding IL-8 compared to untreated static conditions. Of notable interest, analytes that were upregulated by L10H30 (i.e., IL-6, GM-CSF, sICAM-1, CCL3) [Fig. 3(a)] now exhibited lower concentrations than spheroids in untreated static conditions. Other analytes that were increased in trace amounts by L10H30 (i.e., IL-1β, IL-17A) were no longer detectable when treated with Y-27632.

Overall, the disruption of the actin cytoskeleton via Y-27632 downregulated the secretion of immunomodulatory factors from MSC spheroids exposed to L10H30. The secretory levels remained generally unchanged for Y-27632-treated static groups. These data correlate with downregulation of *CTGF* expression with Y-27632 treatment and suggest that cyclic compressive load modulates MSC spheroid immunomodulatory cytokine production via mechanosensing.

### Compression of MSC spheroids influences macrophage polarization

**FIG. 5.**
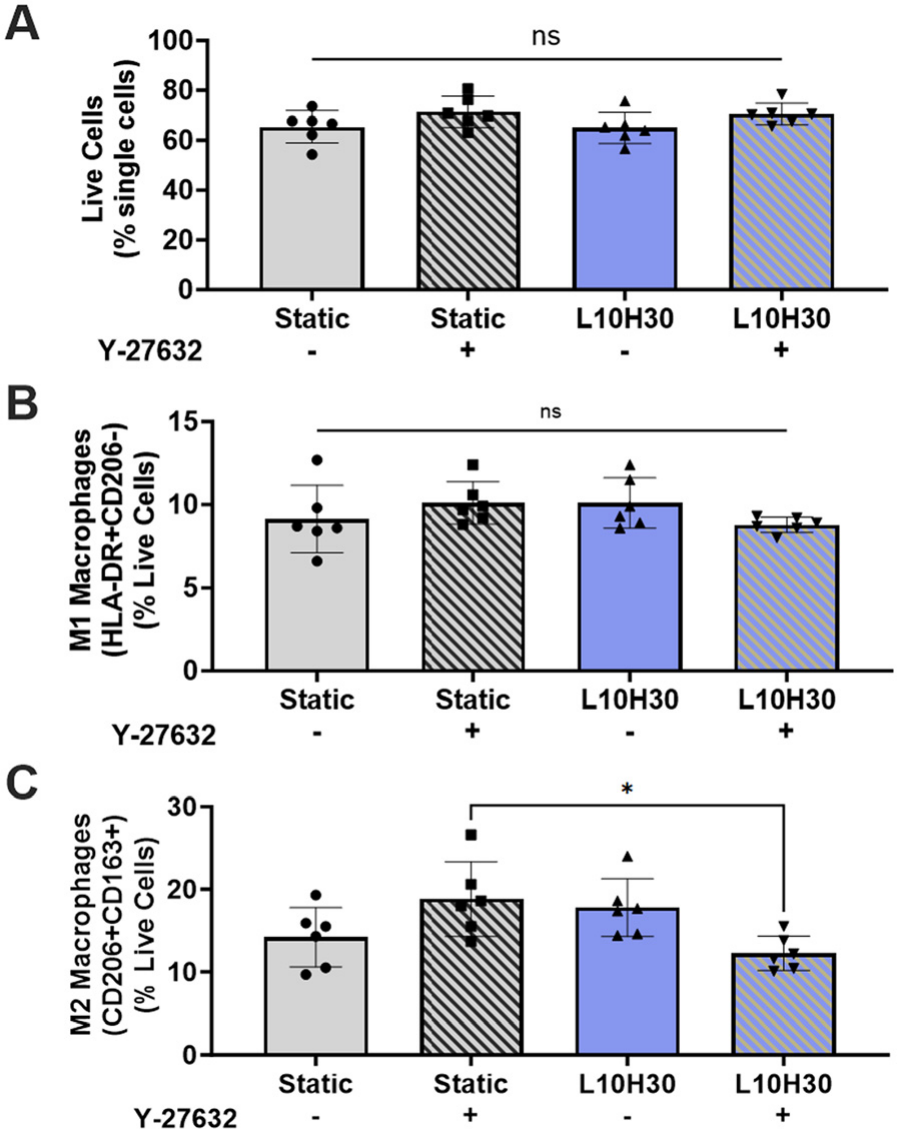
Compressive loading of MSC spheroids modulated macrophage polarization. Flow cytometric analyses of human THP-1 macrophages treated with MSC spheroid conditioned media. Quantification of (a) Live cells, (b) HLA-DR^+^CD206^−^ (M1) macrophages, and (c) CD206^+^CD163^+^ (M2) macrophages. Data are mean ± SD (n = 6). Groups with statistically significant differences (p < 0.05) based on one-way ANOVA are denoted by an asterisk. ns denotes no significance. L10H30 – 10 kPa load, 30 s hold.

Having established changes in the MSC spheroid secretome under compressive loading, we next investigated the functional effects of these changes on immune cell modulation. We treated human THP-1 macrophages with conditioned media and assessed macrophage polarization via flow cytometry. We found no differences in live cells [Fig. 5(a)] or M1 polarized macrophages (HLA-DR^+^CD206-) [Fig. 5(b)] between groups, but we observed significant differences in M2 polarized macrophages (CD206^+^CD163^+^) [Fig. 5(c)]. Interestingly, media from static and L10H30 spheroids were not different, but when treated with Y-27632, M2 macrophages increased for static spheroids but decreased for loaded spheroids. These findings correlate with relative increases in IL-10 between untreated and treated static groups. Additionally, IL-6, a notoriously complex cytokine, can prime macrophages for IL-4-induced M2 polarization.^26^ Given the large relative increase in IL-6 in treated static groups, this also may account for the increase in M2 polarization. Furthermore, we noted relative decrease in anti-inflammatory cytokines (e.g*.,* IL-10, IL-13) between untreated and treated L10H30 spheroids, which correlates with decreased M2 macrophages for those groups.

## DISCUSSION

Cell-based approaches for tissue regeneration and repair frequently utilize MSCs for their potent secretome and are often transplanted into weight-bearing environments for regeneration of bone, cartilage, and muscle. The application of cyclic compressive load potentiates osteogenic and chondrogenic differentiation of MSCs and modulates their secretory profile.^18,20^ However, there is limited understanding of the role of compressive load on the immunomodulatory characteristics of MSCs despite the effectiveness of MSCs in regulating the inflammatory milieu in clinical trials.^27^ A key novelty of this study lies in the use of MSC spheroids, as opposed to monodispersed cells, within an alginate matrix that possesses cell adhesion motifs and viscoelastic characteristics for a more representative tissue-like environment. Our findings demonstrate that the combination of various compressive loads and durations dictate the secretion of pro- and anti-inflammatory cytokines from MSC spheroids. Utilizing a bioreactor system that is compatible with standard incubator conditions enabled precise and continuous mechanical loading, a key factor in the simulation of *in vivo* conditions. Together, these aspects emphasize the study's contribution to advancing therapeutic applications in tissue repair, illuminating the complex interplay between mechanical forces, cellular organization, and biochemical signaling in MSC spheroids.

Biomaterials are often used as cell carriers for cell transplantation or to guide cell behavior.^9^ Alginate is a natural, biocompatible, inert, and easily tunable material that supports MSC spheroid survival, proliferation, and cytokine secretion.^5^ Herein, we entrapped MSC spheroids in RGD-modified alginate to enable essential cell-matrix interaction with the surrounding material for propagation of mechanical signaling, while also increasing trophic factor secretion compared to unmodified alginate.^5^ Over the relatively short time course of this study, we did not observe significant outgrowth of MSCs from the spheroids into the alginate matrix, suggesting that mechanical load was transmitted to cells within the spheroids. Unlike unmodified gels or alginate modified to a significantly higher degree of RGD substitution, these gels support robust cell outgrowth over longer durations.^28,29^

We defined three cyclic compressive regimes with varying stress (L) magnitudes (i.e., 5 or 10 kPa) and hold (H) durations (i.e., 30 or 250 s). Even though the compressive loads we chose for our study are several orders of magnitude less than the compressive strength characteristic of weight-bearing environments such as cortical (130–190 MPa) and trabecular bone (∼50 MPa), these magnitudes allowed us to investigate the effect of different load magnitudes on directing MSC spheroid behavior when entrapped in alginate gels.^30^ While the compressive loads employed do not precisely match those *in vivo*, the research provides critical insights into the impact of mechanical stimulation on the secretion of inflammatory cytokines, which is crucial for regulating tissue development, repair, and regeneration. Cells and tissues are exposed to varied mechanical stresses that influence cell behavior, including the composition of their secretome. Cell-based therapies, such as MSC spheroids, typically have a limited lifespan of a few days to weeks, whereas tissue formation and remodeling occur over longer periods. Future studies that modulate the number of compressive loading cycles by employing shorter hold and rest times would facilitate the study of loading cycle frequency on MSCs. Hold durations of the applied compressive loads were also varied to interrogate the effects on MSC spheroid behavior, which were based on the stress relaxation time of the hydrogel ( $\tau_{1/2}\approx$ 28 s). Cycle times (5 min 30 s) were held consistent for all runs to ensure groups underwent the same number of cycles. Loading regimes with 30 s hold times were accompanied by a 240 s rest period, allowing the gels to fully relax after the applied stress while loading regimes with 250 s hold times only had a 20 s rest period, which did not permit the gels to relax before the next compression was applied.

The secretome of MSC spheroids was dependent on compressive load magnitude and hold time. Using a multiplex cytokine array, we observed that larger compressive loads and shorter durations (L10H30) upregulated pro-inflammatory cytokine production (i.e., IL-6, IL-8), while larger compressive loads and longer durations (L10H250) downregulated anti-inflammatory cytokine production (i.e., IL-10) compared to static conditions. Interestingly, smaller compressive loads and shorter durations (L5H30) enhanced pro-angiogenic cytokine secretion (i.e., VEGF) compared to static conditions. Other cytokines that are key mediators in the inflammatory response, such as IL-1β, GM-CSF, sICAM-1, and CCL3, were also upregulated with application of mechanical load. In addition to their immunomodulatory roles, these analytes have implications in other regenerative processes. For example, IL-6 exerts pro-angiogenic effects demonstrated by increased VEGF expression in a dose-dependent manner when cervical cancer tissues were treated with IL-6.^31^ Similarly, sICAM-1 mediates leukocyte adhesion to endothelial cells and functions as a mediator of angiogenesis by inducing the formation and supporting the survival of microvessels.^32^ Other factors such as GM-CSF promote myeloid cell development and maturation and dendritic cell differentiation while reducing VEGF activity and angiogenesis by inducing monocyte secretion of soluble VEGFR-1, which are advantageous in cancer settings to slow metastasis.^33^ The implications of these analytes on angiogenesis demonstrate the value of compressive loading in influencing a wide range of cell behaviors.

Cells encounter dynamic mechanical environments *in vivo* in scenarios such as bone regeneration or wound healing in which the distribution of stresses is altered by the composition of the surrounding extracellular matrix. This aligns with the changing mechanical demands during bone regeneration, wherein lower strains are initially present, gradually increasing as new tissue forms.^34^ Different compressive loading regimes regulate the expression of mechanosensitive genes through cytoskeletal machinery. The observed changes in actin formation and cytoskeletal reorganization under different loading conditions demonstrate how mechanical cues influence cellular activities, such as secretion of immunomodulatory cytokines and growth factors, and the activation of signaling pathways, including the Hippo pathway, that regulate the response to mechanical loading. In static and L10H30 groups, we observed robust, interconnected F-actin structure, suggesting these conditions facilitated cell elongation, organization, and actin polymerization while L5H30 and L10H250 disrupted actin filament structure. Hence, we focused on static and L10H30 groups to further interrogate the mechanism of action driving the differences we observed in the MSC secretome. Specifically, L10H30 upregulated *CTGF* expression compared to static conditions, indicating that MSCs are responsive to cyclic compressive load through their actin cytoskeleton and the YAP/TAZ pathway. When inhibiting actin polymerization, we noted a downregulation of *CTGF* expression, and we observed a reduction of inflammatory-related cytokines in both static and L10H30 with a greater reduction in the mechanically loaded group. In agreement with this finding, stiffer MSC spheroids exhibited enhanced *YAP1* expression and YAP translocation to the nucleus, a process dependent upon actin polymerization.^35^ While we did not explicitly interrogate YAP translocation due to mechanical loading or abrogation due to Y-27632, future work is warranted to establish the interplay of gene expression, mechanical loading, and YAP translocation on changes in the secretome by stimulated MSC spheroids. Our findings suggest that preconditioning MSC spheroids under specific loading regimes could enhance their regenerative potential. The pharmacological disruption of the cytoskeleton facilitates examination of the interplay between mechanical forces and cellular responses, which is vital for effective tissue engineering strategies. Understanding how loading conditions affect MSC behavior could provide valuable insights for *in vivo* strategies, such as applying mechanical loads through physical therapy or biomechanical devices, to stimulate and guide tissue repair processes. Although it is well established that substrate stiffness influences immune cell recruitment and modulation, our data represent the first, to our knowledge, that under cyclic loading, MSC mechanotransduction pathways may also play a role in MSC-mediated immunomodulation.^36^

The differences we observed in secretory profiles across the various loading regimes opens opportunities for using continuous cyclic compressive loading to prime MSCs for specific therapeutic applications. Though surprising that the L10H30 loading regime did not induce significant functional differences in macrophage polarization compared to static culture, this could imply that compressive loading may influence MSC behavior without disrupting immune modulation on a functional level. This observation merits further investigation to establish the full spectrum of cytokines and chemokines secreted by MSCs under mechanical loading conditions and their impact on different immune cells. Previous *in vivo* studies establish the interplay between macrophages and MSCs, particularly in static environments.^37,38^ However, these studies focus on specific aspects of MSC-immune cell interactions and do not fully capture the broader mechanobiological responses elicited by mechanical loading. They also address static aspects of cell behavior, which might not reflect the dynamic nature of mechanotransduction in MSCs. Our study presents a dynamic and physiologically relevant approach to examine MSC behavior, mimicking the mechanical environment of weight-bearing tissues by the application of compressive load. This approach allows for a more comprehensive understanding of MSC responses, especially in the context of priming macrophages, which play a crucial role in immunomodulation during tissue repair. Our focus on compressive loading, as opposed to static co-culture systems, ensures that our findings are more representative of the complex *in vivo* scenario, enhancing their relevance to therapeutic strategies in regenerative medicine.

Herein, the application of mechanical loading combined with ROCK inhibition by Y-27632 creates a unique cellular environment, potentially altering signaling pathways and cellular responses in a manner distinct from each stimulus independently. Y-27632 impacts cellular function through Rho-associated protein kinases (ROCKs), leading to changes in cytoskeletal phenotype and wound healing. Prior reports suggest that the effects of Y-27632 are influenced by its interaction with specific kinases, rather than solely by the magnitude or duration of mechanical load.^39^ This aligns with our findings of cytokine downregulation under combined mechanical loading and Y-27632 treatment, indicating a complex feedback mechanism that modulates cellular responses. Blebbistatin is another common antagonist to study the interplay of cell contractility and stimulation. In a relevant study examining the effects of blebbistatin derivatives and Y-27632 on non-muscle myosin 2 (NM2) dynamics, Y-27632 accelerated NM2 diffusion in both peripheral and central fibers, while the influence of blebbistatin derivatives were dependent on concentration.^40^ This suggests that these inhibitors affect NM2 diffusion and potentially secretome profiles differently. Our study revealed changes in the secretome that were dependent on load magnitude and duration. While our study did not explore the use of other antagonists or softer matrices, these data provide a valuable framework for future studies to explore how various inhibitors, in combination with mechanical loading, affect MSC behavior and secretome dynamics. Additionally, mechanosensing of substrate stiffness by immune cells may influence mode of migration, cell morphology, secretion of anti- and pro-inflammatory cytokines, and phagocytosis.^36,41^ Future work is needed to elucidate the influence of compressive stress on immune cell recruitment, a critical process during inflammation, as well as the interplay of different MSC spheroid sizes and mechanical load. MSC spheroids were formed at 8000 cells/spheroid to ensure sufficient nutrient and oxygen diffusion, but prior reports established that spheroid diameter influences MSC behavior.^6,42^

We studied the influence of mechanical stimulation using a bioreactor system that provides a continuous physical compressive loading while incubator compatible, which is a substantial limitation of current systems. However, despite the advantages of our model, the application of mechanical load is limited to one direction and therefore does not capture the multidirectional forces cells experience *in vivo*. Furthermore, future investigations are needed to understand how long changes persist in stimulated cells and the impact of mechanical stimulation on cell behavior over longer periods of time. Finally, the viscoelastic nature of alginate guides regenerative cell functions,^43^ and future studies are needed to explore the contributions of viscoelasticity on altering cell behavior in a compressive environment.

## CONCLUSION

We investigated the influence of continuous, physical, compressive load on the immunomodulatory behavior of MSC spheroids. We demonstrated the significant role of load magnitude and duration on modulating the MSC cytoskeleton, secretome, gene expression, and macrophage polarization. Our approach provides a more physiologically relevant model to investigate the effects of compressive loading compared to static, pneumatic, or hydrostatic loading, and offers a potential preconditioning strategy to prime cells for therapeutic applications.

## METHODS

### Cell culture

Human bone marrow-derived MSCs from a single male donor (21-year-old, RoosterBio, Frederick, MD) were expanded without further characterization in standard culture conditions in minimum essential alpha medium (α-MEM; Invitrogen, Carlsbad, CA) supplemented with 10% fetal bovine serum (FBS; Atlanta Biologicals, Flowery Branch, GA) and 1% penicillin/streptomycin (P/S; Gemini Bio-Products, Sacramento, CA) until use at passages 4–5. Media changes were performed every 2–3 days. Human THP-1 monocytes were expanded in RPMI 1640 medium (ATCC Formulation: L-glutamine, HEPES, sodium pyruvate, and high glucose) supplemented with 10% FBS (GenClone, El Cajon, CA) in suspension culture under standard culture conditions until the density reached approximately 1 × 10^6^ cells/ml. Cells were then seeded at 75% confluency and treated with 320 nM phorbol-12-myristate-13-acetate (PMA) for 36 h to induce adherence and differentiation into macrophages.

### Spheroid formation

MSC spheroids were formed using a forced aggregation method.^44^ Briefly, MSCs were pipetted into 1.5% agarose molds in well plates to produce spheroids comprised of 8000 cells and then centrifuged at 500×*g* for 8 min. Plates were maintained in static culture conditions (37 °C, 5% CO_2_, 21% O_2_) for 48 h for spheroid formation in α-MEM.

### Alginate hydrogel synthesis and spheroid encapsulation

PRONOVA Ultra-pure (UP) medium viscosity high-guluronic acid (MVG) sodium alginate (>200 000 g/mol; NovaMatrix, Sandvika, Norway) was oxidized to 1% (w/v).^45^ Alginate was then modified with arginine–glycine-aspartic acid (RGD) using standard carbodiimide chemistry.^28^ The peptide G_4_RGDSP (Peptide 2.0; Chantilly, VA) was added to achieve a degree of substitution (DS) of 2. RGD-modified alginate was then placed in dialysis tubing (3.5 kDa MWCO; ThermoFisher Scientific, Waltham, MA) in a water bath under magnetic stirring for 3 days. Alginate was sterile filtered and lyophilized for 5 days until dry.

Lyophilized RGD-alginate was reconstituted in PBS to obtain a 2% (w/v) alginate solution. MSC spheroids were then entrapped in alginate at 5 × 10^6^ cells/mL and ionically crosslinked with a solution of 200 mM CaCl_2_ and 10 mM BaCl_2_ [Fig. 1(a)]. Briefly, reagents were mixed for 30 s using a positive displacement pipette and cast in 8 mm diameter circular silicone molds. Dialysis membrane (6–8 kDa MWCO; ThermoFisher Scientific) was placed over the molds, and a solution of 200 mM CaCl_2_ and 10 mM BaCl_2_ was added to cover the membrane. After 10 min, the membrane was removed, gels were flipped, and gels were subsequently covered with the same mixture for another 10 min. Gels were allowed to reach swelling equilibrium statically for 24 h in α-MEM before culture in static or dynamic conditions.

### Mechanical characterization of alginate hydrogel

We measured the storage moduli of alginate hydrogels with a Discovery HR2 Hybrid Rheometer (TA Instruments, New Castle, DE) using a stainless steel, cross hatched, 8 mm plate geometry.^12^ An oscillatory strain sweep ranging from 0.004% to 4% strain was performed with an oscillatory angular frequency of 10 rad/s on each gel with an initial axial force of 0.03 N to acquire the linear viscoelastic region (LVR) prior to gel failure. At least five data points were collected for the linear region and averaged to obtain gel shear storage modulus. Gels were measured immediately after gel synthesis (D0) and on day 3 after equilibrating in fully supplemented α-MEM at 37 °C.

The stress relaxation time of alginate hydrogels was determined using an Instron 3345 Compressive Testing System (Norwood, MA). From the stress (normalized) vs time (s) graphs generated, the stress relaxation time was calculated by determining the time for which the stress relaxed to 50% of its initial value ( $\tau_{1/2}$).^12^

### Mechanical loading of alginate hydrogels

We applied compressive mechanical load to cell-laden alginate hydrogels using a MechanoCulture TX (MCTX) bioreactor (CellScale, Ontario, Canada). Mechanical loads were chosen to interrogate the influence of load amplitude on MSC spheroid behavior and were based on the maximum stress the hydrogel could sustain without fracturing. Spheroid-entrapped hydrogels were stimulated with a compressive stress of 5 or 10 kPa and denoted as L5 or L10, respectively. The hold duration of the compressive stress was also varied (i.e., 30 or 250 s) to interrogate these effects on MSC spheroid behavior denoted as H30 or H250, respectively (Table I). Selection of hold and rest times were based on the stress relaxation time of the hydrogel. Times were adjusted to maintain a consistent 5 min 30 s cycle time for all runs. Spheroid-laden alginate hydrogels were allowed to swell for 24 h before loading into the bioreactor. Gels in static culture served as the control. Spheroids were cultured in static or dynamic conditions for 1 or 3 D. The culture media was refreshed every 2 day, and 24 h (1 ml) prior to collection as conditioned media (CM).

### MSC spheroid response to mechanical loading

We assessed cell viability by a live/dead assay per the manufacturer's protocol (ThermoFisher). Metabolic activity was measured after 3 D in static or dynamic culture using an alamarBlue assay (Invitrogen), with fluorescence read at 590 nm. DNA content was quantified via PicoGreen dsDNA assay (Invitrogen). Spheroid entrapped alginate gels were fixed in paraformaldehyde (PFA) at room temperature for 1 h and washed with PBS prior to DAPI (ThermoFisher) and Alexa Fluor Phalloidin 488 staining (ThermoFisher). Spheroids were imaged using confocal microscopy (Leica STELLARIS, Leica Camera AG, Wetzlar, Germany).

The secretory profile of MSC spheroids in static or dynamic culture was characterized with a ProcartaPlex™ human inflammation panel 20-plex kit to measure CD62E (E-selectin), CD62P (P-selectin), GM-CSF, soluble ICAM-1 (sICAM-1), IFN-α, IFN-*γ*, IL-1α, IL-1β, IL-10, IL-12p70, IL-13, IL-17A, IL-4, IL-6, IL-8 (CXCL8), IP-10 (CXCL10), CCL2, CCL3, CCL4, TNFα (CN: EPX200-12185-901; ThermoFisher) and assessed on the Luminex® xMAP 200 (ThermoFisher). The net mean fluorescence intensity (MFI) was measured and calculated for the seven standards and samples, and the data were analyzed using the online ProcartaPlex Analysis Application.^6^ VEGF, IL-6, IL-8, and IL-10 secretions were measured using specific enzyme-linked immunoassay (ELISA) kits according to the manufacturer's protocols (R&D Systems, Minneapolis, MN), as their initial concentrations were out of range of the ProcartaPlex panel. Data were normalized to untreated static or untreated L10H30 and represented as log fold change where 0 indicates no fold change, positive values signify upregulation, and negative values signify downregulation compared to the control.

### Gene expression analysis of MSC spheroids in response to mechanical load

To interrogate the mechanism of how mechanical load may influence the immunomodulatory behavior of MSC spheroids, we treated spheroid-loaded alginate gels with 10 *μ*M Y-27632 dihydrochloride (Y-27632; Bio-Techne, Minneapolis, MN), an inhibitor of ROCK and actin polymerization. We quantified relative gene expression of *CTGF*, a downstream target of Yes-associated protein (YAP), by quantitative real-time polymerase chain reaction (qRT-PCR) after 24 h of treatment. Samples were collected in 1 ml of TRIzol (Invitrogen) and homogenized. RNA was isolated following instructions per the manufacturer. 800 ng of RNA was reverse transcribed using the QuantiTect Reverse Transcription Kit (Qiagen, Hilden, Germany) and normalized to a final concentration of 10 ng/*μ*l. We performed qRT-PCR using *Taq* PCR Master Mix (Qiagen) in a QuantStudio 6 Pro real-time PCR system (ThermoFisher). Human specific primer *CTGF* (Hs00170014_m1) was used (ThermoFisher). Critical threshold values (Ct) were quantified for each gene of interest with a ΔCt value quantified by subtracting the samples Ct value of the *GAPDH* house-keeping gene. The ΔΔCt value was quantified by subtracting the average ΔCt value of respective controls from each sample. Gene expression values were represented as 2^−ΔΔCt^.

### Flow cytometry analysis of MSC spheroids in response to mechanical load

Following differentiation, THP-1 macrophages were rinsed three times with PBS, treated with conditioned media at a 1:1 ratio with basal media, and incubated for 24 h. Media was then replaced with basal media, cells were incubated another 24 h, then collected for flow cytometry. Polarization controls were treated the same, but instead of conditioned media treatments, macrophages were treated with basal media (M0), 200 ng/ml LPS (M1), and 20 ng/ml IL-4 (M2) (data not shown).^46^

Cells were collected with ice cold 2.5 mM EDTA in PBS and gentle scraping, spun down, and resuspended in 37 °C 3% FBS in PBS. Cells were then stained for flow cytometric analysis. Following Fc*γ* receptor blocking (1:40, TruStain FcX, BioLegend), cells were stained with antibodies against CD11b (1:40, eBioscience #47-0118-42), HLA-DR (1:40, eBioscience #48–9956–42), and CD206 (1:33, eBioscience #12-2069-42), and CD163 (1:50, Invitrogen #MA5-17719). Cellular viability was evaluated with fixable Zombie Aqua (1:250, Life Tech). Cells were then fixed with 2% PFA, and analyzed on the flow cytometer (Attune NxT, Thermo Fisher Scientific). Macrophages with an M1 phenotype were characterized by HLA-DR^+^CD206- populations and M2 phenotypes by CD206+CD163+ populations.

### Statistical analysis

Data are derived from a minimum of three independent experiments and presented as means ± standard deviation. Statistical significance was assessed by one-way ANOVA with Tukey's multiple comparisons test or Student's t-test when appropriate. *P* values ≤0.05 were considered statistically significant. Statistical analysis was performed using GraphPad Prism® 9 software (GraphPad Software, San Diego, CA). Significance is denoted by alphabetical letterings. Unless otherwise stated, different letters denote statistical significance between groups.

## SUPPLEMENTARY MATERIAL

See the supplementary material table for details Concentrations of pro- and anti-inflammatory and inflammatory cell recruitment human analytes secreted by MSC spheroids cultured in untreated and Y-27632-treated static and L10H30 conditions.

## Data Availability

The data that support the findings of this study are available from the corresponding author upon reasonable request.
